# Acute-on-chronic liver failure due to thiamazole in a patient with hyperthyroidism and trilogy of Fallot: case report

**DOI:** 10.1186/1471-230X-10-93

**Published:** 2010-08-14

**Authors:** Chuan Shen, Cai-Yan Zhao, Fang Liu, Ya-Dong Wang, Jun Yu

**Affiliations:** 1 Department of Infectious Disease, the Third Affiliated Hospital of Hebei Medical University, Shijiazhuang, China; 2 Institute of Digestive Disease and Department of Medicine and Therapeutics, The Chinese University of Hong Kong, Hong Kong

## Abstract

**Background:**

Thiamazole is a widely used antithyroid agent that has been approved for the treatment of hyperthyroidism. Although thiamazole-induced hepatotoxicity is a main side effect, it may progress to liver failure in a very few cases.

**Case Presentation:**

We described a 24-year-old patient with hyperthyroidism and trilogy of Fallot, who developed liver failure due to thiamazole. Liver biopsy showed intrahepatic cholestasis, mild inflammatory infiltrates, as well as significant fibrosis, indicating both acute and chronic liver injuries. Although a series of potent therapies were given, the patient deceased due to severe liver decompensation.

**Conclusions:**

This case suggests that thiamazole-induced hepatotoxicity in the setting of advanced fibrosis increases the risk of poor outcome. Regular liver function monitoring during thiamazole therapy is therefore important.

## Background

Liver dysfunction is a common complication observed in hyperthyroidism, either as a result of hyperthyroidism and/or the use of antithyroid drugs (ADs). ADs including thiamazole (MMI) and propylthiouracil (PTU) are widely used for treating hyperthyroidism. Despite their relatively safe profiles, both can induce hepatotoxicity presenting as hepatocellular or cholestatic injury [1-6]; however, liver failure has rarely been encountered. Because thyroidectomy and radioiodine are contraindicated in the setting of liver failure, few treatment options are available.

Trilogy of Fallot (TOF) including pulmonic stenosis, interatrial septal defect, and right ventricular hypertrophy is a congenital cardiac disease. Due to the impairment of pulmonary outflow, right-sided congestive heart failure can be observed at its advanced stage, leading to the occurrence of passive liver congestion or development of hepatic fibrosis [7].

Here, we report the first case of a patient with hyperthyroidism and TOF that was recently referred to our hospital for acute-on-chronic liver failure (ACLF) related to MMI.

## Case Presentation

A 24-year-old man was admitted to our hospital due to diarrhea, progressive jaundice, light stool, fatigue and excessive sweating for 18 days. He was diagnosed with TOF at age 3, and had undergone pulmonary valvotomy and closure of the foramen ovale at age 19. The former procedure failed, while the latter was successful. Since then hyperthyroidism was confirmed, and MMI (20 mg per day) was intermittently taken for 1 year because he refused to receive radioiodine therapy. Neither thyroid nor liver function had been regularly monitored during the treatment period or in the following 4 years. MMI (20 mg per day) was continuously taken by himself for 20 days prior to the onset of the symptoms, which, however, did not arouse his alertness, and MMI administration was continued until his admission. He denied use of any other concomitant prescriptions (including non-ADs), over-the-counter drugs or herbal remedies. The patient stated that there was no history of alcohol abuse, food allergies or liver disease.

Physical examination revealed a 112 heart beats per minute with evidences of tremor, generalized icterus, and pigmentation of the skin on the bilateral tibia. Marked carotid pulsation, jugular distension, and an enlarged thyroid gland with vascular murmur were observed. Thrill on palpation and mid-systolic murmur on auscultation were evident in the pulmonary area. A strong collapsing pulse and pistol shot sound was noted in the peripheral arteries. The liver edge and the spleen edge descended 1.5 cm and 3 cm below the costal margin, respectively, and peripheral edema was also noticed.

**Figure 1:**
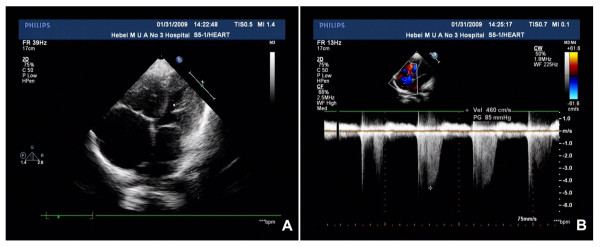
**The color Doppler echocardiographic findings**. A: M-mode and two-dimensional echocardiography shows the right atrial dimensions are 5.71×5.85 cm. B: Continuous wave Doppler shows the tricuspid valve regurgitation velocity is 4.6 m/s and the pressure gradient between right ventricle and right atrium is 85 mmHg.

Laboratory tests revealed the following biochemical results (with the normal range in brackets): ALT 75 U/L (5 to 40), ALP 133 U/L (15 to 130), ALT/ALP ratio 0.56, TBIL 587.9 μmol/L (3.0 to 20.0), DBIL 288.2 μmol/L (2.0 to 6.0), FT4 154.8 pmol/L (7.90 to 14.10), FT3 30.8 pmol/L (3.86 to 6.00), thyroid-stimulating hormone less than 0.01 μIU/ml (0.340 to 5.600), PT 22.2 seconds (9.0 to 12.8), and international normalized ratio 2.3 (0.8 to 1.4). Viral serologies (hepatitis A, B, C, and E viruses; human immunodeficiency virus; Epstein-Barr virus and cytomegalovirus) and autoimmunity markers (antinuclear antibody, antimitochondrial antibody, antineutrophil cytoplasmic antibody, anti-smooth-muscle antibody, and anti-liver-kidney microsome antibody) were all negative. Screening studies were performed to detect genetic and metabolic liver diseases, which excluded hematochromatosis, Wilson's disease, and alpha 1-antitrypsin deficiency. The color Doppler echocardiography showed evidence of severe pulmonary stenosis, right ventricular hypertrophy, tricuspid incompetence with systolic regurgitation, and enlarged right atrium (Figure 1). Moreover, ultrasonography of the abdomen revealed signs of hepatic parenchymal injury, inferior vena cava dilation and splenomegaly.

Drug induced lymphocyte stimulation test for MMI was performed, and the procedure was briefly described as follows: Lymphocytes isolated from the heparinized peripheral blood sample of patient were incubated with serial concentrations of the purified MMI (M8506, Sigma). After that, the proliferative response was assessed by measuring incorporated ^3^H-thymidine uptake. The patient showed a positive result, with the Stimulation Index of 2.19.

**Figure 2:**
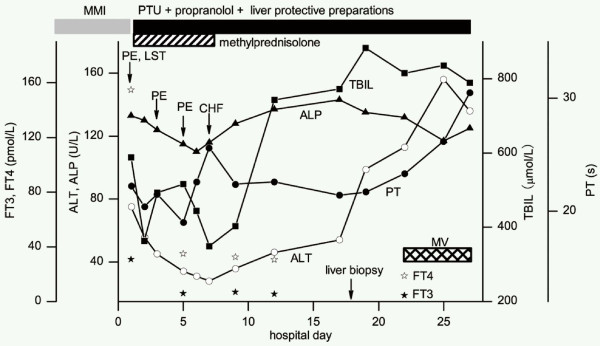
**Serial levels of biochemical parameters during hospitalization**. ALT: alanine aminotransferase; ALP: alkaline phosphatase; TBIL: total bilirubin; PT: prothrombin time; FT3: free triiodothyronine; FT4: free thyroxine; MMI: thiamazole; PTU: propylthiouracil; PE: plasma exchange; CHF: continuous hemofiltration; LST: lymphocyte stimulation test; MV: mechanical ventilation.

**Figure 3:**
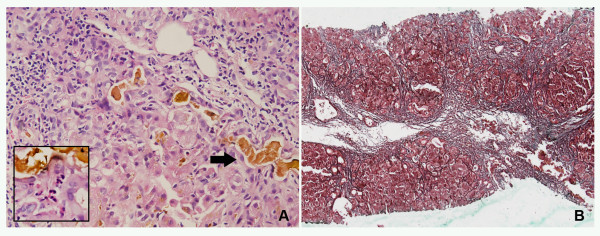
**Histological findings of the liver specimen**. A: Liver biopsy showing intracanalicular bile plugs (black arrow) and enlarged hepatocytes with feathery degeneration, indicative of intrahepatic cholestasis. Moderate inflammatory infiltration, composed of lymphocytes, neutrophils, as well as eosinophils (inset), was presented in portal area (Hematoxylin and Eosin staining, ×400). B: Expanded portal tracts accompanied by significant hepatic fibrosis (Masson Tri-chrome staining, ×100).

MMI was discontinued on admission. The patient was treated with PTU (50 mg 8 hourly) and propranolol (40 mg 3 times daily). Methylprednisolone (40 mg daily) and liver protecting preparation (e.g. diammonium glycyrrhizinate, ursodeoxycholic acid, and ademetionine) were also administrated. The patient underwent three sessions of plasma exchange, and an 8-hour continuous hemofiltration. His general condition, liver functions and other biochemical parameters gradually improved with ALT, ALP, TBIL and PT values, while FT3 and FT4 approximately restored to normal levels; however, two days later, his liver function worsened again (Figure 2). A liver biopsy was performed on day 18 after admission. Pathological findings showed that the portal tracts were expanded by moderate inflammatory infiltration composed of lymphocytes, neutrophils and eosinophils. The portal tracts were surrounded by proliferative cholangioles, and some with bile plugs. Nodular regeneration of the hepatic parenchyma was delimited by loosened fibrous septa. Diffused swelling and enlargement of hepatocytes were detected within hepatic lobules, particularly around central veins. Feathery degeneration was also observed in some hepatocytes, indicative of intracellular cholestasis, but necrosis was absent within portal areas and lobules (Figure 3). During the following 9 days, his liver function profiles progressively deteriorated, and grade II encephalopathy developed. The patient subsequently experienced hypoxemia, hypotension, decreased urinary outflow, and metabolic acidosis. Although mechanical ventilation and intensive therapies were applied, unfortunately, the progression of his medical conditions could not be suspended, and he eventually expired as a result of multiple organ failure on day 27 after admission.

## Conclusions

This patient presented with acute-on-chronic liver failure. After extensive investigations, viral hepatitis, autoimmune liver diseases, Wilson's disease, hemochromatosis, and alpha 1-antitrypsin deficiency were excluded. There was no history of alcohol abuse or exposure to toxic substances. We sought for etiologies of liver injury based on clinical course and suggestive changes in liver histology.

The liver biopsy revealed characteristic features of intrahepatic cholestasis with mild inflammatory infiltrates indicative of acute liver injury. MMI-induced hepatotoxicity was highly suspected, based on: (1) a 1-year history of intermittent use of MMI, which was continuously taken for 20 days before the symptoms onset; (2) the absence of other concomitantly administrated prescriptions, over-the-counter drugs and herbs; (3) the onset of hepatotoxicity was temporally related to the start of MMI therapy before admission; (4) the patient's condition was relatively stable prior to MMI therapy; (5) lymphocyte stimulation test for MMI was positive; and (6) the common causes of liver injury were ruled out. We also assessed the patient using the CIOMS/RUCAM scale, and on the basis of his score 7, he was judged "probable" [8].

Although MMI is generally safe in treating hyperthyroidism, hepatotoxicity has been described as a major side effect with approximately 30 cases described in the English literature [4]. However, there is, up till now, only one case reporting MMI-related acute liver failure in a patient with hyperthyroidism and chronic hepatitis B [9]. Symptom-onset of this adverse effect ranges from 3 days to 5 months following the initiation of MMI therapy [4]. The MMI-induced liver injury usually appears to be a cholestatic process that is not as severe or life-threatening as the hepatocellular damage induced by PTU. Only 6.6% of patients receiving MMI therapy (30 mg per day) showed elevated ALT and AST levels twice the upper of the normal range, compared with 26.9% on PTU (300 mg per day) [10]. The typical features are elevated serum levels of bilirubin, and histological changes of intrahepatic cholestasis with mild periportal inflammation [1,5,6]. Our patient presented with a dramatically high level of bilirubin and the ALT/ALP ratio <2, suggesting the pattern of cholestatic liver injury. Liver biopsy demonstrated obvious intrahepatic cholestasis, proliferative cholangioles with bile plugs, and moderate portal inflammation with eosinophils infiltration, which were consistent with characteristic features of MMI-induced hepatotoxicity in most reported cases.

The mechanism underlying MMI-related liver injury remains unclear, and some researchers proposed that it is most likely an idiosyncratic (hypersensitive) reaction, which is often infrequent and unpredictable [4,6,9]. The onset of symptoms is usually delayed after the start of drug exposure [5,11]. However, it appears more rapidly and severely upon rechallenge of the culprit drug [5,11]. Thus, this may reasonably account for severe hepatotoxicity developed when MMI continuously taken and the phenomenon of lymphocyte proliferation *in vitro*.

The liver histology also indicated advanced hepatic fibrosis and nodular patterns, the hallmark of chronic liver injury, which reflects, in part, continuous acute liver injury extended over time. Due to the absence of other risk factors for causing persistent liver injury, we suspected that the hepatic fibrosis was mostly associated with prolonged hepatocytes hypoxia caused by TOF and hyperthyroidism.

Although central vein/sinusoidal dilation has not been observed on his liver histology, our patient exhibited clinical manifestations (e.g. jugular distension, hepatomegaly, and splenomegaly) and ultrasonographic images (e.g. enlarged right atrium, inferior vena cava, and hepatic vein dilation) that strongly supported right-sided heart failure and passive liver congestion resulting from TOF. Prolonged congestive heart failure can lead to liver damage, mainly because of decreased hepatic blood flow, increased venous pressure, and decreased arterial oxygen saturation [7]. Long-term existence of passive liver congestion, hepatic fibrosis or even cirrhosis may be developed [7].

Hyperthyroidism should, in fact, also reasonably account for chronic liver injury due to quite a long period of non-controlled hyperthyroidism. Thyroid dysfunction has been suggested to perturb liver function, and liver disease, in turn, modulates thyroid hormone metabolism [12]. It is estimated that about 60.5% of patients with hyperthyroidism exhibited at least one hepatic abnormality upon diagnosis [13]. The mechanism is most likely due to hepatocytes hypoxia, a condition caused by a high demand for oxygen, but with no appropriate increase in hepatic blood flow [12]. Previously reported patients with untreated hyperthyroidism can develop a spectrum of pathological changes ranging from focal necrosis to cirrhosis [14]. In addition, hyperthyroidism further promotes the progress of liver injury especially in the setting of congestive heart failure [2].

Indeed, the pre-existing chronic liver disease remained unknown in this patient until the current acute episode, mainly because of patient's asymptomatic state, as well as the paucity of liver function tests due to his poorly medical compliance. However, it is possible that the presence of the underlying chronic liver disease secondary to above medical conditions may have predisposed the patient to MMI-induced hepatotoxicity.

Appropriate management of both hyperthyroidism and liver injury is critical for our patient's condition. Both thyroidectomy and radioiodine are contraindicated in patients with liver failure, limiting the options for the treatment of hyperthyroidism. Switching to PTU has been reported to be effective in 3 cases of MMI-induced hepatotoxicity, because the mechanisms responsible for MMI- and PTU-induced hepatotoxicity differ [5]. PTU, but not MMI, can block the extrathyroidal conversion of thyroxine to triiodothyronine, which may lead to a more rapid reduction in serum triiodothyronine level and to a more rapid resolution of hyperthyroidism symptoms[1,15]. However, alternative ADs was proposed to be a risky approach due to the possibility of cross-reactivity (in up to 50% cases) between two agents [1]. As noted from others, plasma exchange has been used successfully to treat severe hyperthyroidism. The underlying mechanism may be the effective removal of thyroid hormones, which are almost entirely (>99%) bound to plasma proteins [16]. Successful use of steroids has been reported in cases of ADs-related hepatotoxicity; however, whether it benefits accelerating recovery needs further assessment.

Our patient's condition was temporally alleviated under above combination treatment, but promptly worsened again over the following days. We suspect that the deterioration of his liver function might be related to insufficient residual hepatic capacity for regeneration. In addition, the possibility of cross-reactivity between MMI and PTU cannot be fully excluded in this patient, because the progressive elevation of ALT and TBIL levels was observed shortly after the initiation of PTU therapy.

In summary, MMI can induce acute hepatotoxicity, and particularly in the setting of underlying chronic liver disease, the possibility of poor outcome may increase. This case also highlights the importance of regular monitoring of liver function for patients who receive MMI therapy for hyperthyroidism, especially during the first 3 months after initiating therapy. Such closely monitoring will enable early recognition and treatment when hepatic abnormalities occur, and prevent the progression of liver lesions.

## Consent

Written informed consent was obtained from the patient's father for publication of this case report and any accompanying images. A copy of the written consent is available for review by the Editor-in-chief of this journal.

## Abbreviations

ALT: alanine aminotransferase; ALP: alkaline phosphatase; TBIL: total bilirubin; DBIL: direct bilirubin; PT: prothrombin time; FT3: free triiodothyronine; FT4: free thyroxine; MMI: thiamazole; PTU: propylthiouracil.

## Competing interests

The authors declare that they have no competing interests.

## Authors' contributions

ZCY proposed the study, managed the patient, and also critically revised the manuscript. SC, LF and WYD managed the patient, performed the literature search and wrote the paper. YJ provided her advice and revised the manuscript. All authors read and approved the final version.

## Pre-publication history

The pre-publication history for this paper can be accessed here:

http://www.biomedcentral.com/1471-230X/10/93/prepub
